# Assessment of interradicular areas and cortical bone thickness for orthodontic mini-implant placement: a cone-beam computed tomography study

**DOI:** 10.1186/s12903-025-06251-4

**Published:** 2025-05-28

**Authors:** Sanjay Prasad Gupta, Deepa Niroula, Nisha Budha Magar

**Affiliations:** 1 Department of Orthodontics and Dentofacial Orthopedics, Maharajgunj Medical Campus, Institute of Medicine, Tribhuvan University Teaching Hospital, Tribhuvan University, Kathmandu, Nepal; 2 Department of Oral Medicine and Radiology, Maharajgunj Medical Campus, Institute of Medicine, Tribhuvan University Teaching Hospital, Tribhuvan University, Kathmandu, Nepal

**Keywords:** Cone beam computed tomography, Cortical bone thickness, Interradicular dimensions, Mini-implants

## Abstract

**Background:**

Mini-implant has become an accepted and reliable method for providing absolute anchorage in orthodontics. It is known that incorrect implant positioning can increase the failure rates and damage the adjacent tooth roots. This study aimed to assess the interradicular areas and cortical bone thickness for orthodontic mini-implant placement using cone beam computed tomography (CBCT).

**Methods:**

CBCT scans of 100 subjects (42 males, 58 females; mean age of 16.32 ± 5.72 years) were selected consecutively from the records archive of the Department of Orthodontics and Dentofacial Orthopedics, Tribhuvan University Teaching Hospital, Kathmandu, Nepal. A convenience sampling method was employed for selecting the sample. For each interradicular space in the maxilla and the mandible from the central incisor to the second molar; the mesiodistal distance, buccopalatal/lingual thickness, and cortical bone thickness were measured at three different depths from the cementoenamel junction (CEJ), that is at 2 mm, 4 mm, and 6 mm. Descriptive analysis was used to obtain the mean and standard deviation of all the studied measurements. Analysis of variance was used to compare the interradicular dimensions and cortical bone thickness in different regions and at various heights.

**Results:**

There were differences in the interradicular dimensions and cortical bone thickness in different regions and at various heights in both the maxilla and mandible. In the maxilla, the highest mesiodistal distances (3.48 mm, 6 mm apical to CEJ) were between the first molar and second premolar and the highest buccopalatal thickness (14.26 mm, 6 mm apical to CEJ) and buccal cortical thickness (1.92 mm, 4 mm apical to CEJ) existed between the first and second molars and highest palatal cortical thickness (1.77 mm, 6 mm apical to CEJ) were between the first and second premolars. In the mandible, the highest mesiodistal distance (4.74 mm, 6 mm apical to CEJ), buccolingual thickness (13.60 mm, 6 mm apical to CEJ), buccal cortical thickness (3.05 mm, 4 mm apical to CEJ), and lingual cortical thickness (2.59 mm, 4 mm apical to CEJ) were between the first and second molars.

**Conclusions:**

Mesiodistal distance, buccopalatal/lingual thickness, and cortical bone thickness differ in different regions and at various heights in both the maxilla and mandible. Hence, these factors must be considered while assessing the optimal sites for mini-implant placement. The safest interradicular areas in the maxilla were between the first molar and the second premolar, whereas between the first and second molars in the mandible, at 6 mm apical to CEJ.

## Introduction

Miniscrew implants are now recognized as a safe way to provide absolute orthodontic anchorage. Mini-implants have several advantages over the traditional methods of anchorage [1]. When used as temporary anchorage devices, miniscrews have several benefits, including ease of placement and removal, instant loading, versatility in use, absolute anchorage, cost effectiveness, no additional preparation required for placement and require reduced patient cooperation. The restricted interradicular space and worries about harming tooth roots, however, continue to be obstacles to the practical use of these miniscrews.

The palate, the retromolar region in the mandible, the buccal cortical plate in the maxilla and the mandible, and the palatal part of the maxillary alveolar process seem to be the most often used implant locations [2–4]. When selecting mini-implant placement sites, soft-tissue anatomy, inter-radicular distance, sinus morphology, nerve location, buccolingual bone depth, and buccal and lingual cortical thicknesses are some of the crucial parameters to take into account.

As mini-implants enable the clinician to apply simple mechanics for orthodontic tooth movement, the dento-alveolar bone was the most preferred placement site [5]. The specialized literature has often advised placing a miniscrew in the interradicular bone because it facilitates easy placement and removal processes and enables the use of a very straightforward force system [6].

Furthermore, the miniscrew’s small size permits it to be positioned in interradicular space. Because direct force application from the miniscrew head is feasible even in the absence of flap surgery, the inter-radicular alveolar ridge has proven to be a desirable placement site [7]. However, the availability of a small quantity of interradicular bone is necessary for the safe placement of miniscrew implants in the dento-alveolar bone [8]. Furthermore, it has been noted that one significant risk factor for miniscrew implant failure is the closeness of the implants to the dental root [9].

To maintain periodontal health, a minimum of 1 mm of alveolar bone clearance surrounding the screw has been advised. Consequently, for safe miniscrew placement, an interradicular distance greater than 3 mm is required when the miniscrew’s diameter and the minimum alveolar bone clearance are taken into account [6].

The primary stability of the mini-implant and its success mainly depend upon the cortical bone thickness and the safe distance from the vicinity of the roots. It can also be affected by other factors like the angle of screw insertion, screw design and insertion torque [10–11].

Numerous radiographic and anatomical studies have been conducted to precisely determine whether interradicular spaces also known as “Safe zones” are available for the safe insertion of miniscrew implants and to provide an anatomical guide for implant placement between teeth roots [12–15].

Poggio et al. [8] suggested the safest locations in interradicular areas between the first molar and the second premolar, 2–8 mm from the alveolar crest in the region of the posterior maxilla; while in the posterior mandible, it was between the first and second molars.

The mandible appears to have a thicker buccal cortical bone than the maxilla [16–17]. The thickness of the buccal cortical bone in the mandible and maxillary anterior region increases with increasing distance from the alveolar crest, according to the study done by Baumgaertel and Hans on 30 dry skulls [18].

A study by Pradhan et al. assessed the thickness of alveolar bone in the maxillary central incisor using cone beam computed tomography (CBT) and they found thin labial alveolar bone in the maxillary central incisor region [19].

Most of the previous studies have been carried out on a small sample or were limited to the posterior part of the jaws or not assessed at different cut level from the CEJ and mostly used two-dimensional radiography. Moreover, very limited study has been carried out in Nepalese population. Hence, this study aimed to assess the interradicular areas and cortical bone thickness for orthodontic mini-implant placement in both the maxilla and mandible at various levels using CBCT, thereby defining the optimal mini-implant ‘safe zones.’

## Methods

In this analytical cross-sectional study, CBCT scans of 100 subjects (42 males, 58 females; mean age of 16.32 ± 5.72 years) from May 2021 to May 2023 that were already available and required for diagnostic purposes were selected consecutively from the records archive of the Department of Orthodontics and Dentofacial Orthopedics, Tribhuvan University Teaching Hospital, Kathmandu, Nepal. A convenience sampling method was employed for selecting the sample. The consecutive scans meeting the inclusion criteria during that period were included in this study. The sample size was calculated by Altman’s normogram with consideration of alpha error of 0.05 and power of 80%. A total of 100 samples were included to increase the power of the study.

The inclusion criteria were full CBCT having clear images. Patients with overlapping crowns or roots of adjacent teeth, periodontal disease, severe ectopic eruption, missing teeth (excluding third molars), and mixed dentition or incomplete crown eruption were excluded from this study.

Before conducting this study, ethical approval was obtained from the institutional review committee of the Institute of Medicine [Ref.291 (6–11) E^2^,079/080].

The 3D CBCT images were obtained by Rainbow TM CBCT machine (Dentium, Korea) using standard volume protocol at 8 MA, 94 kvp, voxel size of 300 μm and 16 cm x10cm field of view. Orthogonal tomographic images were constructed using Sentium Rainbow TM image viewer (Dentium, Korea). The principal investigator was involved in taking all the measurements. Four weeks later, the same investigator remeasured the 20 randomly selected cases to test for intraobserver reliability. The intra-class correlation coefficient value of 0.93 showed excellent reliability. To minimize measurement errors produced from non-standardized head postures, all images were oriented using a standardized protocol- the palatal plane aligned parallel to the horizontal axis supplied by the software, and the nasal septum was parallel to the vertical axis.

### Measurements

**Fig. 1:**
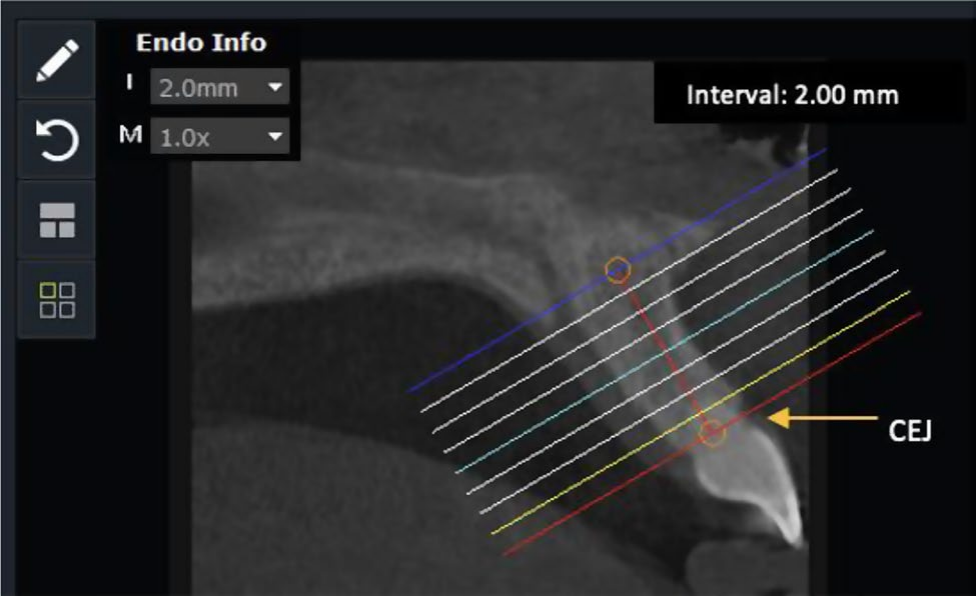
Measurements at an interval of 2 mm from the cementoenamel junction (CEJ)

For each interradicular space in the maxilla and the mandible, from the central incisor to the second molar, the following measurements were done at three different depths from the cementoenamel junction (CEJ), that is at 2 mm, 4 mm, and 6 mm (Fig. 1). These distances were measured using the millimetric ruler provided in the software.

**Fig. 2:**
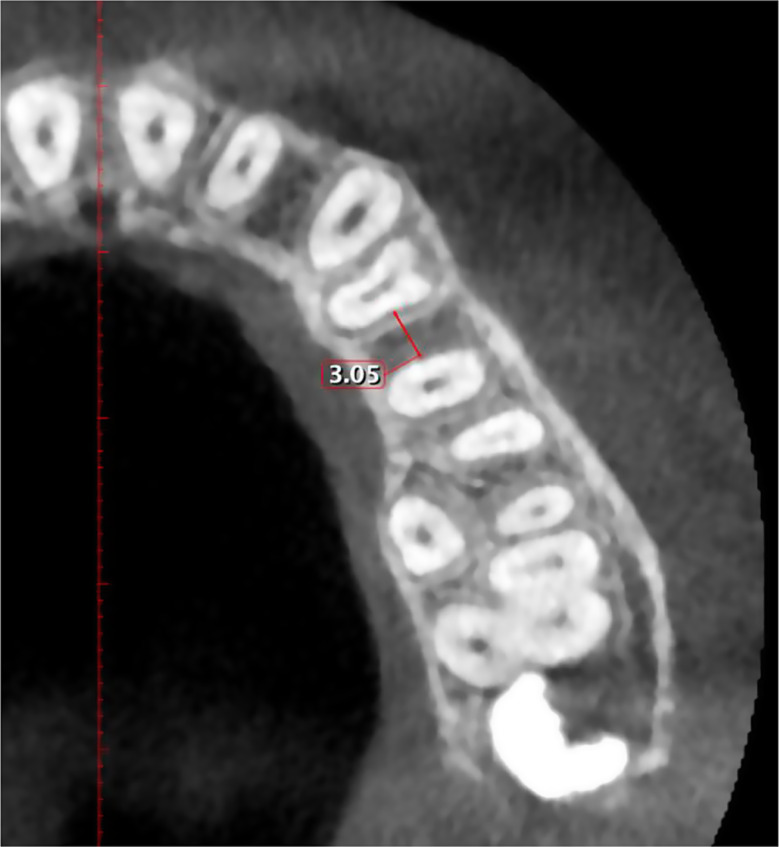
Measurement of mesiodistal distance between maxillary 1st and 2nd premolar at the level of 4 mm apical to the CEJ

- Mesiodistal distance (Inter-radicular distance): In axial sections, measurements were taken at the smallest distance between two adjacent teeth at 2, 4 and 6 mm apical to CEJ (Fig. 2).

**Fig. 3:**
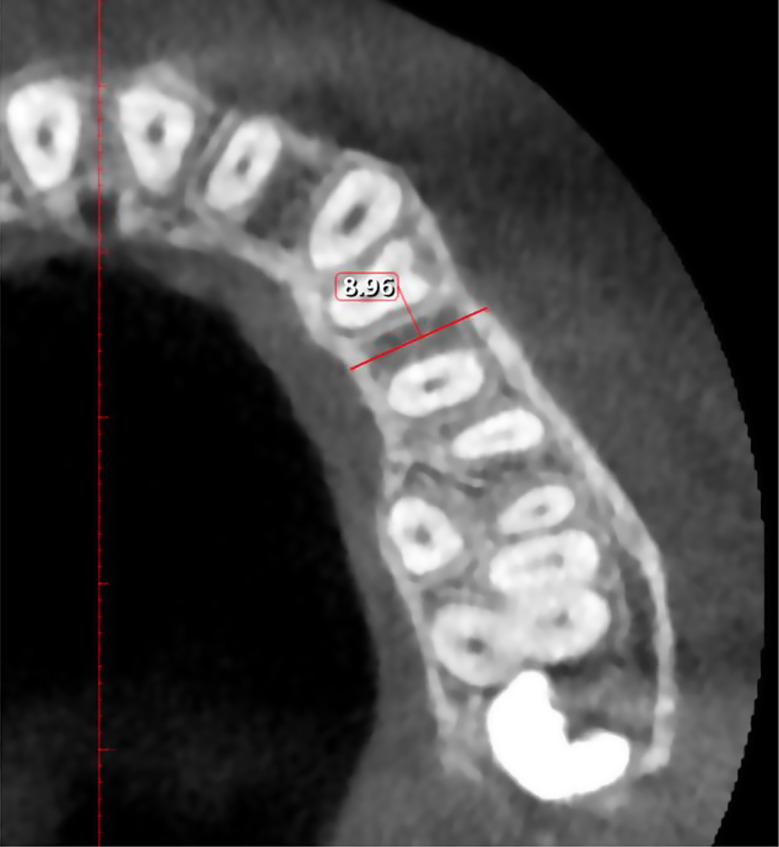
Measurement of bucco-palatal thickness between maxillary 1st and 2nd premolar at the level of 4 mm apical to the CEJ

Bucco-palatal/lingual thickness (Bone thickness): In axial section, measured from the outermost point on the buccal side to the outermost point on the palatal/lingual side at the middle of two adjacent teeth at 2, 4, and 6 mm apical to CEJ (Fig. 3).

**Fig. 4:**
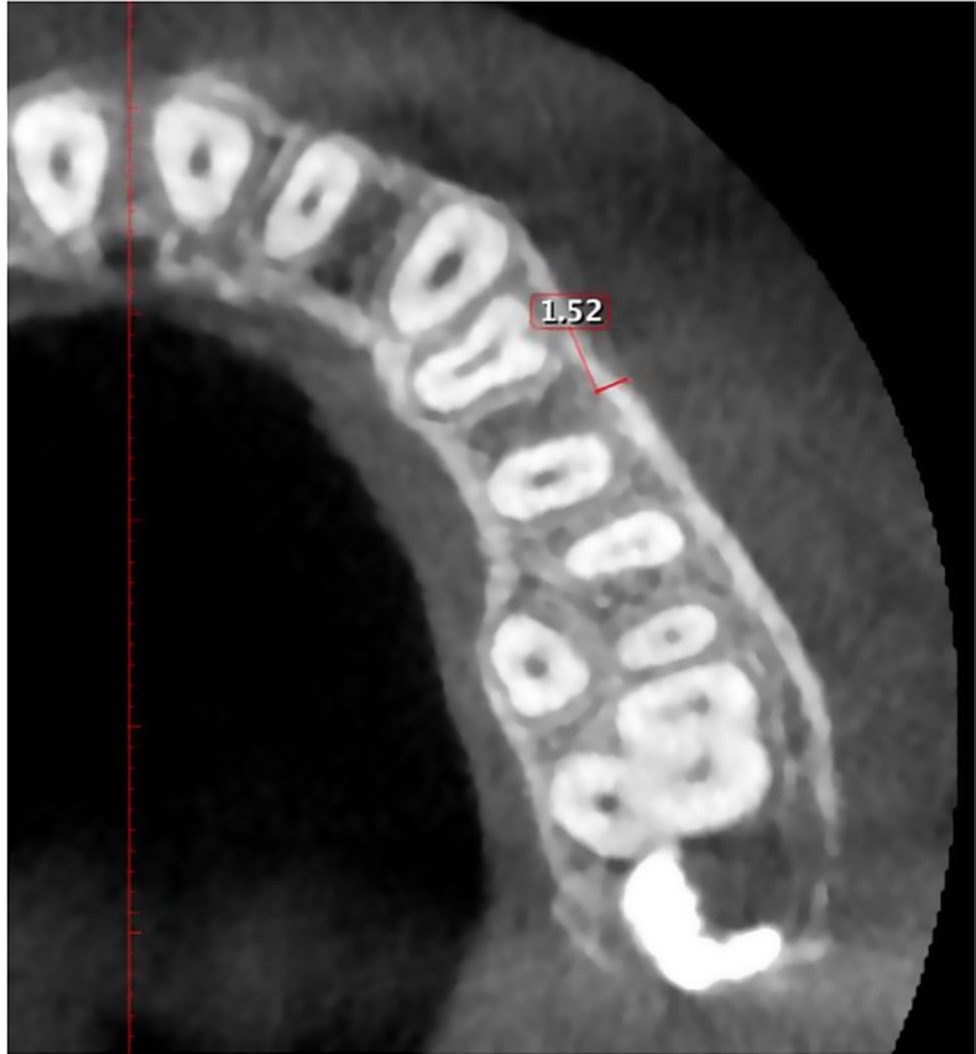
Measurement of buccal cortical bone thickness between maxillary 1st and 2nd premolar at the level of 4 mm apical to the CEJ

**Fig. 5:**
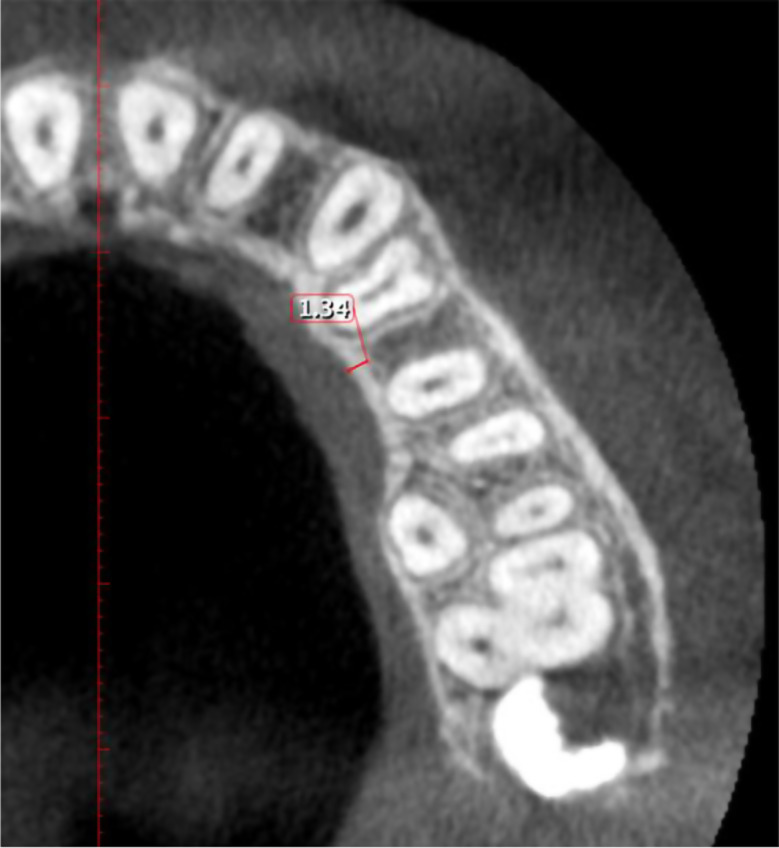
Measurement of palatal cortical bone thickness between maxillary 1st and 2nd premolar at the level of 4 mm apical to the CEJ

Cortical bone thickness: Buccally and lingually/palatally, the distance between internal & external aspects of the cortex in the middle of two adjacent teeth at 2, 4, and 6 mm apical to CEJ (Figs. 4 and 5).

Data obtained were transferred to MS-excel sheet. The data were double entered, verified, and analyzed statistically using SPSS Statistics software version 21.0 (Armonk, NY: IBM Corp.) with confidence level set at 95% (*P* < 0.05).

Descriptive analysis was used to obtain the mean and standard deviation (SD) of all the studied measurements. Shapiro-Wilk test was carried out to test for the normality. It showed normal distribution, hence parametric test was used for further evaluation. Analysis of variance (ANOVA) was used to compare the interradicular dimensions and cortical bone thickness in different regions and at various heights in both the maxilla and mandible.

## Results

In this study, one hundred three-dimensional (3D) images of subjects were selected, out of which 42 were male and 58 were female. The mean age of the patients was 16.32±5.72 years.

For each interradicular space in the maxilla and the mandible from the central incisor to the second molar; the mesiodistal distance, buccopalatal/lingual distance, and cortical bone thickness were measured at three different depths from the cementoenamel junction (CEJ), that is at 2 mm, 4 mm, and 6 mm which is depicted in Tables 1, 2, 3 and 4. Whereas, the comparison of various parameters at different cut levels and in different areas using the ANOVA test is depicted in Table 5.

In the maxilla, the mesiodistal distance ranges from 1.58 to 3.48 mm. The lowest (1.58 mm) was between the central and lateral incisor at 2 mm apical to CEJ and the highest (3.48 mm) was between the second premolar and first molar at 6 mm apical to CEJ (Table 1). This distance increases gradually as we move apically from the CEJ. There was a significant difference in mesiodistal dimension at different cut levels (*p* = 0.005) and in different areas from the central incisor to the second molars (*p* = 0.0003) (Table 5).

In the mandible, the mesiodistal distance ranges from 1.8 to 4.74 mm. The lowest (1.8 mm) was between the central and lateral incisor at 2 mm apical to CEJ and the highest (4.74 mm) was between the first and second molars at 6 mm apical to CEJ (Table 2). This distance increases gradually as we move apically from the CEJ. There was a significant difference in mesiodistal dimension at different cut levels (*p* = 0.0045) and in different areas from the central incisor to the second molars (*p* < 0.001).

In the maxilla, the bucco-palatal thickness ranges from 7.86 to 14.26 mm. The lowest (7.86 mm) was between the central and lateral incisor at 2 mm apical to CEJ and the highest (14.26 mm) was between the first and second molars at 6 mm apical to CEJ (Table 1). These distances show consistent increases in most places as we move apically from the CEJ. There was a significant difference in bucco-palatal thickness at different cut levels (0.0007) and in different areas from the central incisor to the second molars (*p* < 0.001).

In the mandible, the bucco-lingual thickness ranges from 5.56 to 13.60 mm. The lowest (5.56 mm) was between two central incisors at 4 mm apical to CEJ and the highest (13.60 mm) was between the first and second molars at 6 mm apical to CEJ (Table 2). These distances show consistent increases in most places as we move apically from the CEJ. There was no significant difference in buccolingual thickness at different cut levels (*p* = 0.122) but a significant difference in different areas from the central incisor to the second molars (*p* < 0.001).

In the maxilla, the buccal cortical bone thickness ranges from 1.22 to 1.92 mm. The lowest (1.22 mm) was between two central incisors at 2 mm apical to CEJ and the highest (1.92 mm) was between the first and second molars at 4 mm apical to CEJ (Table 3). There was no significant difference in buccal cortical thickness at different cut levels (0.294) but a significant difference in different areas from the central incisor to the second molars (*p* = 0.0013). Whereas, palatal cortical bone thickness ranges from 1.15 to 1.77 mm. The lowest (1.15 mm) was between two central incisors at 2 mm apical to CEJ and the highest (1.77 mm) was between the first and second premolars at 6 mm apical to CEJ. There was a significant difference in palatal cortical thickness at different cut levels (0.001) and in different areas from the central incisor to the second molars (*p* = 0.0008).

In the mandible, the buccal cortical bone thickness ranges from 1.23 to 3.05 mm. The lowest (1.23 mm) was between the central incisor and lateral incisor at 4 mm apical to CEJ and the highest (3.05 mm) was between the first and second molars at 4 mm apical to CEJ (Table 4). There was a significant difference in buccal cortical thickness at different cut levels (*p* = 0.028) and in different areas from the central incisor to the second molars (*p* < 0.001). Whereas the lingual cortical bone thickness ranges from 1.51 to 2.59 mm. The lowest (1.51 mm) was between the central incisor and lateral incisor at 4 mm apical to CEJ and the highest (2.59 mm) was between the first and second molars at 4 mm apical to CEJ. There was a significant difference in lingual cortical thickness at different cut levels (*p* = 0.003) and in different areas from the central incisor to the second molars (*p* < 0.001).

In the maxilla, the highest mesiodistal distance was between the first molar and second premolar; the highest buccolingual thickness and buccal cortical thickness were between the first and second molars and palatal cortical thickness was between the first and second premolars.

In the mandible, the highest mesiodistal distance, buccolingual thickness, buccal cortical thickness and lingual cortical thickness were between the first and second molars.

**Table 1:** Measurements of mesiodistal distance and bucco-palatal thickness at different areas in different cross-sections of the maxilla

		Site						
Cut level	Parameters	1–1	1–2	2–3	3–4	4–5	5–6	6–7
		Mean±SD	Mean±SD	Mean±SD	Mean±SD	Mean±SD	Mean±SD	Mean±SD
2-mm cut	Mesio-distal distance (MD; mm)	2.09±0.58	1.58±0.24	2.27±0.84	1.73±0.55	2.41±0.28	2.42±1.21	1.97±0.83
	Bucco-palatal thickness (BP; mm)	8.45±1.35	7.86±0.94	8.15±1.20	8.21±0.72	9.57±1.43	10.97±1.43	11.72±0.45
4-mm cut	Mesio-distal distance (MD; mm)	2.39±0.73	1.81±0.29	2.43±0.83	1.94±0.48	2.74±0.76	2.52±0.50	2.36±0.97
	Bucco-palatal thickness (BP; mm)	8.71±2.00	8.77±1.75	9.10±1.20	9.56±1.28	10.60±1.94	11.36±1.96	13.92±1.02
6-mm cut	Mesio-distal distance (MD; mm)	2.57±0.62	2.13±0.37	2.87±0.85	2.00±0.48	2.71±0.65	3.48±0.54	2.54±1.00
	Bucco-palatal thickness (BP; mm)	8.49±2.28	8.53±1.48	9.36±1.65	9.91±1.56	9.80±1.75	11.56±0.97	14.26±0.97

**Table 2:** Measurements of mesiodistal distance and buccolingual thickness at different areas in different cross-sections of mandible

		Site						
Cut level	Parameters	1–1	1–2	2–3	3–4	4–5	5–6	6–7
		Mean±SD	Mean±SD	Mean±SD	Mean±SD	Mean±SD	Mean±SD	Mean±SD
2-mm cut	Mesio-distal distance (MD; mm)	2.20±0.53	1.8±0.15	2.21±0.91	2.88±0.58	2.72±0.76	3.64±1.36	3.69±0.44
	Bucco-lingual thickness (BL; mm)	6.05±1.68	6.65±1.44	7.33±1.32	8.1±0.75	7.78±0.57	8.71±1.08	12.11±2.02
4-mm cut	Mesio-distal distance (MD; mm)	2.48±0.61	2.30±0.29	2.7±0.40	2.80±0.66	2.91±0.62	3.83±1.03	3.34±0.43
	Bucco-lingual thickness (BL; mm)	5.56±1.62	6.18±1.45	7.15±2.14	8.10±1.45	8.75±1.11	8.97±1.05	13.33±2.02
6-mm cut	Mesio-distal distance (MD; mm)	2.3±0.50	2.29±0.49	2.53±0.65	3.38±0.96	3.23±0.71	4.09±1.28	4.74±0.37
	Bucco-lingual thickness (BL; mm)	6.25±1.70	6.88±1.50	7.50±2.18	8.08±2.07	8.81±1.37	10.27±1.88	13.60±2.00

**Table 3:** Measurements of buccal cortical thickness and palatal cortical thickness at different areas in different cross-sections of the maxilla

		Site						
Cut level	Parameters	1–1	1–2	2–3	3–4	4–5	5–6	6–7
		Mean±SD	Mean±SD	Mean±SD	Mean±SD	Mean±SD	Mean±SD	Mean±SD
2-mm cut	Buccal cortical thickness (BC; mm)	1.22±0.29	1.22±0.31	1.37±0.28	1.32±0.20	1.45±0.22	1.63±0.17	1.34±0.12
	Palatal cortical thickness (PC; mm)	1.15±0.25	1.21±0.37	1.48±0.21	1.31±0.18	1.27±0.26	1.42±0.30	1.43±0.14
4-mm cut	Buccal cortical thickness (BC; mm)	1.22±0.26	1.31±0.21	1.45±0.17	1.46±0.17	1.39±0.24	1.47±0.27	1.92±0.79
	Palatal cortical thickness (PC; mm)	1.31±0.41	1.37±0.35	1.52±0.36	1.47±0.24	1.65±0.33	1.61±0.54	1.76±0.13
6-mm cut	Buccal cortical thickness (BC; mm)	1.27±0.42	1.23±0.48	1.47±0.16	1.62±0.20	1.45±0.25	1.47±0.32	1.55±0.13
	Palatal cortical thickness (PC; mm)	1.26±0.38	1.34±0.30	1.54±0.40	1.54±0.16	1.77±0.47	1.66±0.29	1.65±0.20

**Table 4:** Measurements of buccal cortical thickness and lingual cortical thickness at different areas in different cross-sections of the mandible

		Site						
Cut level	Parameters	1–1	1–2	2–3	3–4	4–5	5–6	6–7
		Mean±SD	Mean±SD	Mean±SD	Mean±SD	Mean±SD	Mean±SD	Mean±SD
2-mm cut	Buccal cortical thickness (BC; mm)	1.33±0.24	1.41±0.16	1.54±0.31	1.65±0.36	1.78±0.40	2.09±0.53	2.59±0.58
	Lingual cortical thickness (LC; mm)	1.57±0.11	1.89±0.21	1.81±0.08	1.83±0.10	1.96±0.24	1.93±0.11	1.98±0.19
4-mm cut	Buccal cortical thickness (BC; mm)	1.32±0.25	1.23±0.13	1.51±0.19	1.56±0.11	1.62±0.24	1.93±0.32	3.05±0.48
	Lingual cortical thickness (LC; mm)	1.65±0.25	1.51±0.20	1.83±0.20	1.9±0.13	2.31±0.39	2.18±0.33	2.59±0.21
6-mm cut	Buccal cortical thickness (BC; mm)	1.44±0.29	1.41±0.24	1.55±0.35	1.94±0.30	1.79±0.25	2.36±0.33	2.99±0.61
	Lingual cortical thickness (LC; mm)	1.64±0.37	1.89±0.18	1.91±0.35	2.11±0.48	2.28±0.51	2.09±0.18	2.34±0.15

**Table 5:** Comparison of various parameters at different cut levels and in different areas using the ANOVA test

Parameters	At different cut levels (*p*-value)	In different areas from central incisor till second molar (*p*-value)
Maxilla		
Mesio-distal distance	0.005*	0.0003*
Bucco-palatal distance	0.0007*	< 0.001*
Buccal cortical thickness	0.294	0.0013*
Palatal cortical thickness	0.001*	0.0008*
Mandible		
Mesio-distal distance	0.0045*	< 0.001*
Bucco-lingual distance	0.122	0.001*
Buccal cortical thickness	0.028*	< 0.001*
Lingual cortical thickness	0.003*	< 0.001*
(*p < 0.05 = Statistically significant)		

## Discussion

Orthodontists have traditionally focused on attaining a suitable anchorage, although traditional approaches to anchorage have been linked to several issues [20]. According to Newton’s third law of motion, the reactive forces cause tooth movement in the opposite direction in regions where active forces are applied. Although it has certain drawbacks, adding more teeth to the anchoring unit helps reduce unfavorable tooth motions [21]. Extraoral appliances can occasionally be used to improve anchorage, but their effectiveness is dependent on the patient’s compliance and they are a little unesthetic.

Kuhlberg and Burston proposed a way to tackle the problem of anchorage [22]. They suggested bodily movement in the anchorage unit by tipping movement in the movement unit since tipping movement is simpler than bodily movement. Patients who are semi-edentulous or whose teeth should only move in one direction cannot use this procedure. These issues led to the introduction of mini-implants, which act as an anchorage inside the oral cavity without the need for teeth [23].

Numerous recommendations have been made to insert a miniscrew in the interradicular bone since it is simple to place, remove, and apply force [7, 24]. The amount of interradicular bone that is available is necessary for the safe placement of miniscrew implants in the dento-alveolar bone [8, 25]. For safe miniscrew placement, an interradicular gap of at least 3 mm is advised [6]. The inter-radicular spaces and cortical bone thickness between the teeth should be assessed for the miniscrews’ safety and stability, and consequently, their success, as the mini-implants pass through both soft and hard tissues.

Research on the accuracy of 3D measures using CBCT images has demonstrated that they are more accurate than 2D measurements and closer to reality [26, 27]. Using cone beam computed tomography (CBCT), this study assessed the interradicular space and cortical bone thickness at various levels from the CEJ towards the apex.

Previous studies used the reference measurements taken from the alveolar crest [1, 8]. However, in clinical practice, the alveolar crest is not directly visible and that could be affected by different periodontal problems. Several studies have correlated the alveolar crest level with the height of the attached gingiva to provide a more clinically applicable reference point [15, 28] and it is also advisable to place the mini-screws in areas of attached gingiva [29, 30]. Hence, in this study, we have used CEJ as the starting point for the measurements and the maximum level selected to be 6 mm from the CEJ, which is in the area of attached gingiva.

The maxilla showed the greatest buccopalatal thickness between the first and second molars at 6 mm apical to CEJ, and the largest mesiodistal dimensions between the first molar and the second premolar. The findings demonstrated that the ideal location for mini-implant placement in the maxilla is the interradicular area between the first molar and second premolar, which is 6 mm above the CEJ. These findings are consistent with those of previous studies [13, 17, 27]. At 6 mm apical to CEJ, the mandible’s first and second molars had the greatest buccolingual thickness and mesiodistal distance.

As we travel apically away from the CEJ, the mesiodistal distance (interradicular distance) steadily increases. Our results indicate that the best location for mini-implant placement in the mandible is the interradicular space from the canine up to second molar, which is 6 mm from the CEJ, and from second premolar up to second molar, which is 4 mm from the CEJ, as the interradicular measurements were greater than 3 mm [6].

The thickness of the buccal cortical bone in our investigation ranges from 1.22 to 1.92 mm and palatal cortical bone thickness varies between 1.15 and 1.77 mm in the maxilla. Likewise in the mandible, the thickness of the buccal cortical bone varies between 1.23 and 3.05 mm and the thickness of the lingual cortical bone varies between 1.51 and 2.59 mm. The maxilla’s buccal and palatal cortical thicknesses were maximum between the first and second molars and first and second premolars, respectively. On the other hand, the mandible’s first and second molars had the greatest buccal and lingual cortical thickness.

In contrast to studies by Cho and Park [17], Lim et al. [30], and Moslemzade et al. [31], ours did not demonstrate a consistent increase in cortical bone thickness as we moved apically from CEJ. Studies by and Baumgaertel and Hans [18] and Kim et al. [32] also showed no consistent increase in cortical bone thickness as we move apically from CEJ. It may be due to regional bone remodeling or alveolar process anatomy.

According to earlier studies, the miniscrew needs at least 1 mm of cortical bone thickness to have adequate initial stability following insertion [33, 34]. In each location we studied, we found cortical bone thickness of greater than 1 mm.

In both the maxilla and the mandible, the bucco-lingual/palatal thickness consistently increased as we moved apically from the CEJ, which is consistent with the findings of other studies [12, 35].

In the present study, we chose only one side of the arch that meets the inclusion criteria for the CBCT evaluation, as the previous studies suggested no significant difference between both sides of the jaw [27, 36]. In this study, results were not compared between males and females as the sample distribution between males and females was not equal. In addition, Lim et al. reported no significant differences in root proximity between males and females [30].

In this study, we have excluded the broader age ranges to minimize their effects on cortical bone thickness and interradicular dimension. In this study, we have not considered the effect of age on these dimensions, although age may have a potential impact on these findings.

Another limitation of this study is that we did not compare the measurements in different skeletal classes of malocclusions. Multicenter, collaborative, prospective studies in diverse populations, with larger sample sizes and the linking of CBCT metrics to implant stability and patient outcomes, are recommended for a comprehensive assessment.

## Conclusions

The mesiodistal distance, buccopalatal/lingual thickness, and cortical bone thickness differ in different regions and at various heights in both the maxilla and mandible. Hence, these factors must be considered while assessing the optimal sites for mini-implant placement. The safest interradicular areas in the maxilla were between the first molar and the second premolar, whereas between the first and second molars in the mandible, at 6 mm apical to CEJ.

## Data Availability

The datasets used and analysed during the current study are available from the corresponding author on reasonable request.

## Abbreviations

ANOVA: Analysis of variance
CEJ: Cemento-enamel junction
CBCT: Cone-beam computed tomography
3D: Three-dimensional

## References

[1] Monnerat C, Restle L, Muchab JN. Tomographic mapping of mandibular interradicular spaces for placement of orthodontic miniimplants. Am J Orthod Dentofac Orthop. 2009;135(4):428.e1– 9; Discussion 428–29. 10.1016/j.ajodo.2008.12.003 19361724

[2] Park HS, Kwon OW, Sung JH. Nonextraction treatment of an open bite with micro screw anchorage. Am J Orthod Dentofac Orthop. 2006;130:391–402. 10.1016/j.ajodo.2005.07.014 16979500

[3] Kanomi R. Mini-implant for orthodontic anchorage. Am J Orthod Dentofac Orthop. 1997;31:763–7. 9511584

[4] Park HS, Lee SK, Kwon OW. Group distal movement of teeth using microscrew implant anchorage. Angle Orthod. 2005;75:602–9. 16097229 10.1043/0003-3219(2005)75[602:GDMOTU]2.0.CO;2

[5] Aranyawongsakorn S, Torut S, Suzuki B, Suzuki EY. Insertion angulation protocol for Miniscrew implant placement in the Dentoalveolar area. J Dent Assoc Thai. 2007;57:285–97.

[6] Chaimanee P, Suzuki B, Suzuki EY. Safe zones for Miniscrew implant placement in different dentoskeletal patterns. Angle Orthod. 2011;81:397–403. 21261491 10.2319/061710-111.1 PMC8923544

[7] Lee KJ, Joo E, Kim KD, Lee JS, Park YC, Yu HS. Computed tomographic analysis of tooth-bearing alveolar bone for orthodontic Miniscrew placement. Am J Orthod Dentofac Orthop. 2009;135:486–94. 10.1016/j.ajodo.2007.05.019 19361735

[8] Poggio PM, Incorvati C, Velo S, Carano A. Safe zones: a guide for Miniscrew positioning in the maxillary and mandibular arch. Angle Orthod. 2006;76:191–7. 16539541 10.1043/0003-3219(2006)076[0191:SZAGFM]2.0.CO;2

[9] Kuroda S, Yamada K, Deguchi T, Hashimoto T, Kyung HM, Takano-Yamamoto T. Root proximity is a major factor for screw failure in orthodontic anchorage. Am J Orthod Dentofac Orthop. 2007;131:S68–73. 10.1016/j.ajodo.2006.06.017 17448389

[10] Leo M, Cerroni L, Pasquantonio G, Condo SG, Condo R. Temporary anchorage devices (TADs) in orthodontics: review of the factors that influence the clinical success rate of the mini-implants. La Clin Therapeutica. 2016;167(3):e70–77. 10.7417/CT.2016.1936 27424513

[11] Turkyilmaz I, Tözüm TF, Tumer C. Bone density assessments of oral implant sites using computerized tomography. J Oral Rehabil. 2007;34(4):267–72. 17371564 10.1111/j.1365-2842.2006.01689.x

[12] Fayed MM, Pazera P, Katsaros C. Optimal sites for orthodontic mini-implant placement assessed by cone beam computed tomography. Angle Orthod. 2010;80(5):939–51. 20578867 10.2319/121009-709.1 PMC8939012

[13] Sangroula S, Mahto RK, Mishra RK, Shrestha S, Kafle D. Comparison of inter-radicular distance and buccal cortical bone thickness in class I and class II skeletal malocclusion patterns. Orthod J Nepal. 2022;12(2):33–42.

[14] Yang L, Li F, Cao M, Chen H, Wang X, Chen X, Gao W, Petrone JF, Ding Y. Quantitative evaluation of maxillary interradicular bone with cone-beam computed tomography for bicortical placement of orthodontic mini-implants. Am J Orthod Dentofac Orthop. 2015;147(6):725–37. 10.1016/j.ajodo.2015.02.018 26038077

[15] Vasoglou G, Apostolopoulos K, Vasoglou M. Optimal buccal site for Mini-Implant placement on attached gingiva of posterior maxilla: A CBCT study. Appl Sci. 2023;13(12):7099.

[16] Deguchi T, Nasu M, Murakami K, Yabuuchi T, Kamioka H, Takano-Yamamoto T. Quantitative evaluation of cortical bone thickness with computed tomographic scanning for orthodontic implants. Am J Orthod Dentofac Orthop. 2006;129:e7217–72112. 10.1016/j.ajodo.2006.02.026 16769488

[17] Park J, Cho HJ. Three-dimensional evaluation of interradicular spaces and cortical bone thickness for placement and initial stability for microimplants in adults. Am J Orthod Dentofac Orthop. 2009;136:e3141–31412. 10.1016/j.ajodo.2009.01.023 19732658

[18] Baumgaertel S, Hans B. Buccal cortical bone thickness for mini-implant placement. Am J Orthod Dentofac Orthop. 2009;136:230–5. 10.1016/j.ajodo.2007.10.045 19651353

[19] Pradhan S, Shrestha R, Gorkhali RS, Koirala PK. Assessment of labial alveolar bone thickness in maxillary central incisor using cone beam computed tomography. J Nepal Soc Perio Oral Implantol. 2021 Jan-Jun;5(9):2–6.

[20] Melsen B, Bosch C. Different approaches to Anchorage: a survey and an evaluation. Angle Orthod. 1997;67(1):23–30. 9046396 10.1043/0003-3219(1997)067<0023:DATAAS>2.3.CO;2

[21] Quinn RS, Yoshikawa DK. A reassessment of force magnitude in orthodontics. Am J Orthod. 1985;88(3):252–60. 3862348 10.1016/s0002-9416(85)90220-9

[22] Burstone CJ. The segmented arch approach to space closure. Am J Orthod. 1982;82(5):361–78. 6961808 10.1016/0002-9416(82)90185-3

[23] Kuhlberg AJ, Burstone CJ. T-loop position and anchorage control. Am J Orthod Dentofac Orthop. 1997;112(1):12–8. 10.1016/s0889-5406(97)70268-3 9228836

[24] Kanomi R. Mini-implant for orthodontic anchorage. J Clin Orthod. 1997;31(11):763–7. 9511584

[25] Lascala CA, Panella J, Marques MM. Analysis of the accuracy of linear measurements obtained by cone beam computed tomography (CBCT-NewTom). Dentomaxillofac Radiol. 2004;33(5):291–4. 15585804 10.1259/dmfr/25500850

[26] Hilgers ML, Scarfe WC, Scheetz JP, Farman AG. Accuracy of linear temporomandibular joint measurements with cone beam computed tomography and digital cephalometric radiography. Am J Orthod Dentofac Orthop. 2005;128(6):803–11. 10.1016/j.ajodo.2005.08.034 16360924

[27] Hu KS, Kang MK, Kim TW, Kim KH, Kim HJ. Relationships between dental roots and surrounding tissue for orthodontic mini screw installation. Angle Orthod. 2009;79(1):37–45. 19123704 10.2319/083107-405.1

[28] Baumgaertel S. Hard and soft tissue considerations at mini-implant insertion sites. J Orthod. 2014;41(1):S3–7. 25138363 10.1179/1465313314Y.0000000104

[29] Melsen B. Mini-implants: where are we? J Clin Orthod. 2005;39:539–47. 16244412

[30] Lim WH, Lee SK, Wikesjo ¨ UM, Chun YS. A descriptive tissue evaluation at maxillary interradicular sites: implica- tions for orthodontic implant placement. Clin Anat. 2007;20:760–5. 17584877 10.1002/ca.20513

[31] Moslemzade S, Kananizadeh Y, Nourizadeh A, Sohrabi A, Panjnoosh M, Shafiee E. Evaluation of cortical bone thickness of mandible with cone beam computed tomography for orthodontic mini implant installation. Adv Biosci Clin Med. 2014;2(2):55–63.

[32] Kim HJ, Yun HS, Park HD, Kim DH, Park YC. Soft-tissue and cortical-bone thickness at orthodontic implant sites. Am J Orthod Dentofac Orthop. 2006;130:177–82. 10.1016/j.ajodo.2004.12.024 16905061

[33] Dalstra MCP, Melsen B. Load transfer of miniscrews for orthodontic anchorage. J Orthod. 2004;1:53–62.

[34] Motoyoshi M, Yoshida T, Ono A, Shimizu N. Effect of corticalbone thickness and implant placement torque on stability oforthodontic mini-implants. Int J Oral Maxillofac Implants. 2007;22:779–84. 17974113

[35] Jassim WR, Al-nakib LH. Evaluation of interradicular cortical bone thickness for orthodontic Miniscrew implant placement using cone beam computed tomography. Iraqi Dent J. 2016;38(1):28–35.

[36] Atson CS, Sandra QU, Murillo LM, Laila AM, Márcio M. Direct and tomographic dimensional analysis of the inter-radicular distance and thickness of the vestibular cortical bone in the parasymphyseal region of adult human mandibles. Br J Oral Maxillofac Surg. 2012;50(4):350–5. 21636186 10.1016/j.bjoms.2011.05.004

